# Removal of Arsenic Oxyanions from Water by Ferric Chloride—Optimization of Process Conditions and Implications for Improving Coagulation Performance

**DOI:** 10.3390/ijerph18189812

**Published:** 2021-09-17

**Authors:** Muhammad Ali Inam, Rizwan Khan, Kang-Hoon Lee, Young-Min Wie

**Affiliations:** 1 Institute of Environmental Sciences and Engineering (IESE), School of Civil and Environmental Engineering (SCEE), H-12 Campus, National University of Sciences and Technology (NUST), Islamabad 44000, Pakistan; ainam@iese.nust.edu.pk; 2 Department of Chemical Engineering, Quaid-e-Awam University of Engineering, Science and Technology (QUEST), Nawabshah 67480, Pakistan; rizwansoomro@quest.edu.pk; 3 Department of Civil and Environmental Engineering, Hanyang University, Seoul 04763, Korea; 4 Department of Materials Engineering, Kyonggi University, Suwon 16227, Korea; supreme98@kyonggi.ac.kr

**Keywords:** adsorption, arsenic (III–V), coagulation, ferric chloride, pollutant, uptake index, water treatment

## Abstract

The chronic ingestion of arsenic (As) contaminated water has raised significant health concerns worldwide. Iron-based coagulants have been widely used to remove As oxyanions from drinking water sources. In addition, the system’s ability to lower As within the maximum acceptable contamination level (MCL) is critical for protecting human health from its detrimental effects. Accordingly, the current study comprehensively investigates the performance of As removal under various influencing factors including pH, contact time, temperature, As (III, V) concentration, ferric chloride (FC) dose, and interfering ions. The optimum pH for As (V) removal with FC was found to be pH 6–7, and it gradually decreased as the pH increased. In contrast, As (III) removal increased with an increase in pH with an optimum pH range of 7–10. The adsorption of As on precipitated iron hydroxide (FHO) was better fitted with pseudo-second order and modified Langmuir–Freundlich models. The antagonistic effect of temperature on As removal with FC was observed, with optimum temperature of 15–25 °C. After critically evaluating the optimum operating conditions, the uptake indices of both As species were developed to select appropriate an FC dose for achieving the MCL level. The results show that the relationship between residual concentration, FC dose, and adsorption affinity of the system was well represented by uptake indices. The higher FC dose was required for suspensions containing greater concentration of As species to achieve MCL level. The As (V) species with a greater adsorption affinity towards FHO require a relatively smaller FC dose than As (III) ions. Moreover, the significant influence of interfering species on As removal was observed in simulated natural water. The author hopes that this study may help researchers and the drinking water industry to develop uptake indices of other targeted pollutants in achieving MCL level during water treatment operations in order to ensure public health safety.

## 1. Introduction

In past years, arsenic (As) contamination of drinking water supplies has received substantial worldwide attention due to its high toxicity and human health risks. The oral intake of As contaminated drinking water may cause lung, bladder, and liver cancers, skin lesions, muscular weaknesses, neurological disorders and loss of appetite [1]. Therefore, As and its compounds are classified as a primary interest pollutant by various governments and agencies including the European Union (EU) and the US Environmental Protection Agency (USEPA) [2]. In addition, As is categorized as a class 1 carcinogen and its maximum acceptable contamination level (MCL) of 10 µg/L is recommended in drinking water by the World Health Organization (WHO) [3]. The water streams in the Stampede and Slate Creek watersheds in Alaska, USA presented higher As concentration i.e., 720 µg/L [4]. Moreover, higher As contamination was also observed in groundwater near mining sites in Slovakia (285 µg/L) [5]. A high level of As contamination (up to 500 µg/L) in the surface and groundwater of Sindh and Punjab provinces of Pakistan have been reported [6]. Since large populations within countries rely on groundwater sources the provision of As-free drinking water with an MCL of 10 μg/L or less, as specified by the Pakistan Environmental Protection Agency (Pak-EPA), is critical [7]. For this reason, it is essential to treat As polluted drinking water sources properly in order to protect public health.

Several treatment technologies including adsorption, coagulation-flocculation-sedimentation (C/F/S), electrochemical methods, ion exchange, oxidation, phytoremediation, and bioremediation have been applied to remove toxic contaminants from water [8]. Among them, C/F/S and electrocoagulation (EC) processes have shown efficient As removal performance with greater removal of As (III) ions in the case of EC than C/F/S process [9]. It has been suggested that the involvement of oxidation of trivalent As to pentavalent As followed by surface complexation resulted in higher As (III) removal during the EC process [9]. However, the drinking water industries are preferentially operating their conventional C/F/S treatment units worldwide. Conventional treatment with iron-based coagulants has been recognized as one of the most promising techniques for the removal of As species from natural water reservoirs [2,10,11,12]. For instance, ferric chloride (FC) coagulant has been found to be cost-effective and efficient in the remediation of various heavy metal ions from water. An earlier study showed better pentavalent (As (V)) species removal performance than trivalent (As (III)) species with initial contaminant concentration of 100 µg/L, still the system was unable to achieve an MCL in most aqueous matrices [11]. Similarly, our previous study [2] showed high adsorption capacity of FC for pentavalent As species as compared with trivalent As ions with high contaminant loading of 1000 µg/L. It was notable that the coagulation parameters including pH, FC dose, and initial As concentration affected the treatment efficiency of the system [2,10]. Further, the coexistence of other interfering species including phosphate, organic matter etc. hinders the formation of iron hydroxide precipitates, thus increasing the As concentration above permissible level in such solutions [11,12]. The release of both iron (Fe) species and As oxyanions in such drinking water supplies can cause a significant impact on human health. Therefore, it will be worthy to optimize the operating conditions of treatment units in order to reduce the mobility of both toxicants within the MCL in water.

As noted above, previous research studies [2,13] mainly focused on the maximum adsorption ability of the treatment system. In doing so, the misinterpretation of results was most obvious since the treatment efficiency of the system in achieving an MCL of As oxyanions was ignored. The lack of universal and absolute indices for the evaluation and comparison of treatment efficacy is an obvious misapprehension in the production of previously presented outcomes [2,14,15,16]. The standard method, particularly in the case of As remediation, is to evaluate adsorption isotherms following jar tests as verification of the coagulant’s affinity to reduce pollutant concentration. Such diagrams indicate the adsorption affinity of the iron hydroxide precipitates via concentration of As removed per concentration of iron hydroxides precipitates. In contrast, researchers generally considered maximum As adsorption ability as the main criteria in determining efficiency of the conventional treatment unit. In such a case, the extremely high residual concentrations were ignored and misinterpreted in literature [2,14,16]. The percentage of elimination (residual-to-initial concentration ratio) is used in several other circumstances to justify higher As removal affinity. This type of assessment often relates to high residual As from point source needs without considering the amount of coagulant supplied in the treatment process. For example, lowering concentration of As species from 5 mg/L to 0.1 mg/L using C/F/S process corresponds to 98% removal, but residual As concentration still exceed 10 times the MCL for drinking water. In contrast, a decrease in initial As concentration of 50 µg/L by 80% in response of coagulant could be considered permissible for drinking water supplies. Therefore, an uptake index (q_MCL_) for As oxyanions should be developed by the removal performance which shows a remaining concentration of pollutant equal to the MCL. Our recent study [17] attempts to derive a q_MCL_ index for antimony (Sb) oxyanions after the propagation of MCL to the adsorption isotherm. The developed q_MCL_ index was based on modified Langmuir–Freundlich isotherm with Sb contaminant loading up to 1000 µg/L using FC as a coagulant [17]. Similarly, the q_MCL_ index for As species may provide a direct estimate of coagulant efficiency based on the needs of the designed process under common experimental conditions. Therefore, it will be imperative to derive the q_MCL_ index for As species and explore its effectiveness in environmental relevant conditions.

In general, the reported research efforts dealing with As removal from drinking water demonstrates the lack of a generalized method for performance evaluation of the overall system. In addition, there are serious concerns about the methods used to validate their As removal potential from drinking water. There is a need of developing quality indices for As oxyanions under common experimental conditions to enhance the efficiency of the C/F/S method. Thus, the objective of this study is: (i) to investigate the effect of various operating parameters i.e., pH, contact time, temperature, As (III, V) concentration, FC dose and interfering ions on As removal in order to optimize process conditions; (ii) to accurately derive the relation between uptake index, residual concentration and adsorption affinity of As oxyanions under varying concentrations (100, 500, and 1000 µg/L) using FC as a coagulant and; (iii) to help researchers and drinking water industries to develop pollutant uptake indices for improving the drinking water quality in order to ensure human health and safety.

## 2. Materials and Methods

### 2.1. Chemicals and Reagents

The analytical grade reagents including sodium arsenate dibasic heptahydrate (Na_2_HAsO_4_.7H_2_O) and arsenic trioxide (As_2_O_3_) were supplied by Sigma Aldrich (St. Louis, MO, USA). The chemical reagents; sodium bicarbonate (NaHCO_3_), sodium nitrate (NaNO_3_), sodium fluoride (NaF), sodium hydroxide (NaOH), calcium chloride (CaCl_2_∙2H_2_O), magnesium sulphate (MgSO_4_∙7H_2_O), sodium dihydrogen phosphate (NaH_2_PO_4_∙H_2_O), sodium silicate (NaSiO_3_.5H_2_O), hydrochloric acid (HCl), nitric acid (HNO_3_), and ferric chloride hexahydrate (FeCl_3_.6H_2_O) were purchased from local suppliers.

### 2.2. Preparation of Stock Solutions and Synthetic Test Samples

The freshly produced deionized (DI) water from Millipore water purification system (Milli-Q, Millipore Co., Bedford, MA, USA) was used in stock solutions and test samples preparation. In consideration of contamination risks, all sample vessels and collection glassware were initially rinsed using 15% HNO_3_ standard solution and DI water. The pentavalent and trivalent As stock suspensions (0.1 g/L) were synthesized by solubilizing Na_2_HAsO_4_.7H_2_O and As_2_O_3_ in DI water and 1 mol/L NaOH solution, respectively. The 0.1 mol/L ferric chloride (FC) coagulant stock suspension was prepared by solubilizing FeCl_3_.6H_2_O in DI water. The test samples were then synthesized by diluting the desired quantity of As (III) and As (V) stock solutions in DI water for each experimental run.

### 2.3. Jar Test Procedure and Experimental Conditions

The jar tester was used to carry out C/F/S experiments which includes multiple paddles (Model: SJ–10, Young Hana Tech Co., Ltd., Gyeongsangbuk-Do, S. Korea) at ambient temperature. A series of coagulation experiments were conducted under various ranges of pH (4–10), temperature (25 °C), As (III, V) concentration (1000 µg/L) and FC dose (0.1 m mol/L) respectively. Under such conditions, the characteristics of FHO precipitates were also measured using Zetasizer (Malvern, Worcestershire, UK). The experiments were also conducted to investigate the effect of contact time (0–25 min), temperature (15–35 °C), optimum pH, initial As (III, V) concentration (1000 µg/L) and FC dose (0.1 m mol/L) respectively. Following these experiments, the FC dose of (0.01 to 0.3) m mol/L was spiked in synthetic test samples containing different As (III, V) concentrations (100, 500, and 1000 µg/L) under optimum pH and temperature. In all experiments, pH was adjusted using 0.1 mol/L HCL and 0.1 mol/L NaOH solutions with a calibrated pH meter (HACH: Model HQ40d, Loveland, CO, USA), while temperature was adjusted to desirable condition and monitored using digital thermometer. The C/F/S experiments were conducted in following sequence: 3 min rapid agitation (140 rpm); 20 min slow mixing (40 rpm); sedimentation time (30 min) and; aliquot collection by filtering supernatant via 0.45-micron glass filter [2,12]. The aliquots were then analyzed for residual As (III, V) and Fe(III) concentrations using inductively coupled plasma optical emission spectrometry (ICP-OES: Model Varian, Agilent technologies, Santa Clara, CA, USA). The chemical modelling software Visual MINTEQ was used to draw speciation diagram of Fe (III), As (III), and As (V) species. The experiments were carried out twice and the relative mean deviation were reported.

### 2.4. Mathematical Analysis

The mathematical expressions used for reporting precipitated iron hydroxide (FHO): C_s_ (mol/L) (Equation (1)), removal: R (%) (Equation (2)) and adsorption capacity: q_e_ (g/mol) (Equation (3)) are shown below:

(1) $$\mathrm{FHO}:{\;C}_{s}={\;I}_{o}-I_{e}$$

(2) $$\mathrm{Removal}:R=\frac{\left(C_{o}-C_{e}\right)}{C_{o}}\times 100\%$$

(3) $$\mathrm{Adsorption}\;\mathrm{capacity}:{\;q}_{e}=\frac{C_{o}-C_{e}}{C_{s}}$$

where C_o_ and C_e_ (µg/L) represents the initial and residual As (III, V) concentration in test samples, respectively; and I_o_ and I_e_ (mol/L) indicate initial and residual concentration of Fe species in suspensions, respectively. In order to understand the sorption kinetics of As species onto FHO, concentrations of As (III, V) and FHO was observed for time interval of 28 min for temperature of 15–35 °C. The obtained sorption data was then fitted with non-linear Lagergren pseudo-first order (PFO) (Equation (4)) and pseudo-second order (PSO) (Equation (5)) [18,19], as shown below:

(4) $$\mathrm{PFO}:\;q_{t}=q_{e}-e^{\ln\left(q_{e}\right)-k_{1}t}$$

(5) $$\mathrm{PSO}:\;q_{t}=\frac{k_{2}q_{t}^{2}t}{1+k_{2}q_{e}t}$$

where *q_e_* and *q_t_* (g/mol) represents adsorption affinity of As oxyanions at equilibrium and various time intervals, respectively; t (min) indicate contact time; and *k*_1_ (1/min) and *k*_2_ (mol/g min) indicate rate constants of PFO and PSO models, respectively. In order to further explore the application of sorption data, experiments were carried out using FC dosages of 0.01 to 0.3 m mol/L and As (III, V) concentrations of 100, 500, and 1000 µg/L under optimum process conditions. It is worth mentioning that previous studies [2,10] preferred Langmuir–Freundlich isotherms for plotting their data points, however, our recent study [20] indicated the usefulness of a modified Langmuir–Freundlich (mL-F) isotherm model. It was observed that Langmuir–Freundlich-type adsorption can be effectively simulated by an mL-F model [20]. Therefore, the obtained experimental data points were fitted with mL-F isotherm model using Equation (6) [18]:

(6) $$\mathrm{mL}-F\;\mathrm{equation}:\;q_{e}=\frac{q_{m}k_{L.F}C_{e}^{n}}{1+k_{L.F}C_{e}^{n}}$$

where *q_m_* (g/mol) refers to the maximum sorption affinity, *n* is the heterogeneity index, and *k_L.F_* (L/µg) indicate constant of dissociation with low values presenting Freundlich adsorption properties and high values show Langmuir sorption characteristics of As (III, V) oxyanions on iron hydroxide. The popular mathematical software Origin Pro 9.0 (OriginLab, Massachusetts, MA, USA) was used to plot all experimental results.

### 2.5. Natural Water Sample

In order to extend the application of uptake index for As species, the natural waters matrix, such as the National Sanitation Foundation (NSF) standard was used as reference. The NSF was prepared by dissolving 0.178 mg NaH_2_PO_4_∙H_2_O, 252 mg NaHCO_3_, 147 mg CaCl_2_∙2H_2_O, 128.3 mg MgSO_4_∙7H_2_O, 12.14 mg NaNO_3_, 2.21 mg NaF, and 70.6 mg NaSiO_3_.5H_2_O in 1L DI water. Table 1 presents corresponding cations and anions concentrations as below:

## 3. Results and Discussion

### 3.1. Influence of pH on ζ-Potential of Precipitates and As Removal

**Figure 1:**
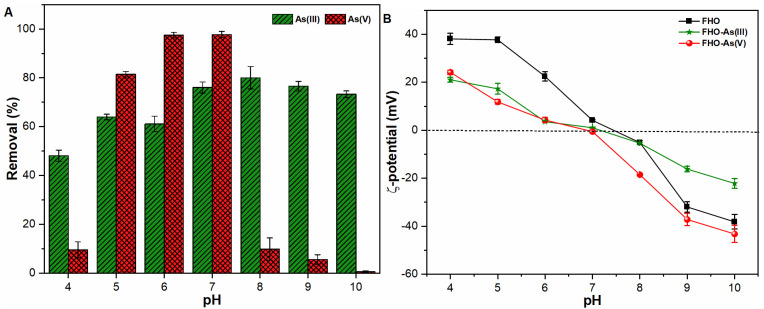
(**A**) Removal of As (III–V) species from water and (**B**) ζ-potential of FHO precipitates across a pH range of 4–10, temperature of 25 °C, FC dosage of 0.1 m mol/L and initial As loading of 1000 µg/L.

The removal of metal ions from water bodies is a function of solution pH owing to different characteristics of various metals. Accordingly, Figure 1A indicates the effect of pH on the removal performance of As oxyanions using FC as a coagulant. A significant decline in As (V) removal was observed under alkaline conditions. Such observation may be attributable to the dissolution of FHO precipitates (see supplementary information (SI): Figure S1A) as a result of a build up of negative charge associated with Fe (III) as well as As (V) species. This ultimately results in strong electrostatic repulsion between both species, thereby inducing strong electron density [21,22,23]. The lowering of the energy barrier resulted in the breakage of the Fe–O bond leading to dissolution of Fe atoms or Fe complexes, hence increasing As concentration in such solutions [2]. As shown in Figure 1A, the significant decrease of As (V) species was observed across a pH range of 8–10. In contrast, the removal of As (III) species increased with increasing pH with an optimum pH range of 7–10. Such an observation may be related to the fact that the first dissociation constant of As (III) species is 9.22 and that H_3_AsO_3_ is a Lewis base and can interact with amphoteric FHO over a broad pH range [24,25]. However, the high As (V) removal was observed in a pH range of 5–7 which may be ascribed to the electrostatic attraction between positively charged Fe(III) complexes and negatively charged pentavalent As species in such suspensions [2,11,26]. At pH range 5–7, it was observed that the dominant form of Fe (III) species was Fe (OH)_2_^+^, while As (V) species exist as H_2_AsO_4_^-^ and HAsO_4_^−2^ under such a pH environment (Figure S1B,C). In general, both As species showed better removal performance under neutral pH conditions, hence a pH of 7 was used for subsequent studies.

**Figure 2:**
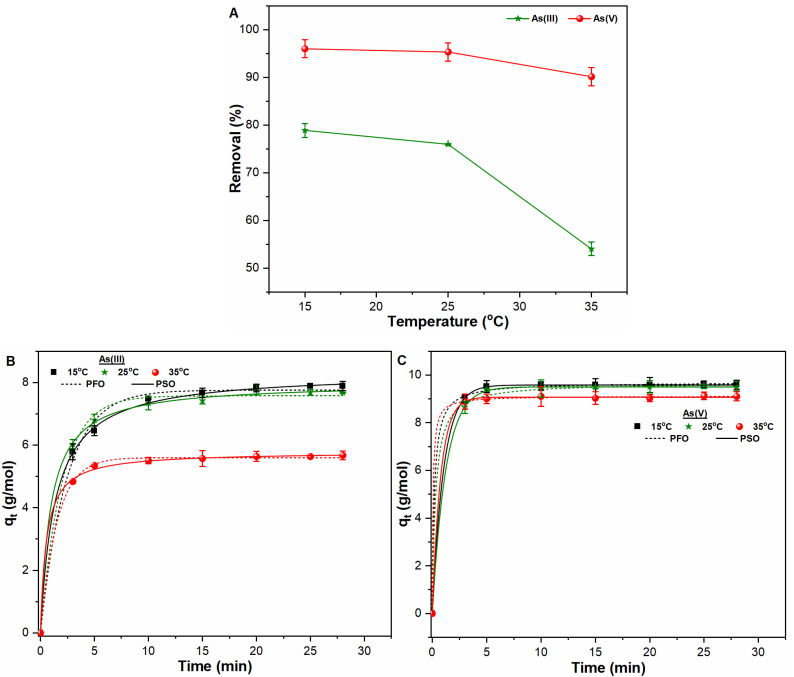
(**A**) Removal of As oxyanions under varying temperature (15–35 °C); and kinetic removal along with fitting parameters of (**B**) Trivalent As; and (**C**) Pentavalent As species from water at pH 7, FC dosage of 0.1 m mol/L, initial As (III, V) concentration (1000 µg/L), and contact time (0–28 min).

In order to further explore the insights into the removal characteristics of As oxyanions using FC coagulant, Figure 2B presents the surface charge of flocs before and after interaction with As (III, V) species. Under acidic pH conditions, the presence of As (V) species brought the ζ-potential values of FHO precipitates close to isoelectric point, and hence showed better removal performance, when compared with As (III) species. In contrast, the presence of negatively charged As (V) species shifted the ζ-potential values of FHO precipitates towards a more negative trajectory under alkaline conditions (Figure 2B). The strong interactive behavior of As (III) with FHO at alkaline pH conditions resulted in less of a decrease in ζ-potential values, therefore, better As (III) removal was achieved in a pH range of 7–10 (Figure 1A,B). In general, the results obtained suggested that pH exhibits a primary influence on the surface properties and stability of FHO precipitates in the presence of As (III, V) species, and thus affect the removal performance of both ions during chemical coagulation process.

### 3.2. Influence of Contact Time and Temperature on As Removal

Contact time and temperature are critical for improving the coagulant’s efficiency in removing the pollutants from water. Therefore, to explore the influence of contact time and temperature on the removal process, the adsorption ability and applicability of kinetic models were evaluated for both As oxyanions in water. For such a purpose, the FHO formation was continuously monitored under varying temperature conditions (see SI: Figure S2A,B). It was observed that an increasing temperature resulted in considerable decrease in FHO formation in As (III) suspensions (Figure S2A), thereby affecting the removal process. Figure 2A indicates the influence of temperature on the removal of both As oxyanions during the FC coagulation process. It is notable that the temperature had a detrimental impact on As (III) removal which may be related to the relatively lesser FHO formations in As (III) suspensions (Figure 2A and Figure S2A). Such removal behavior may also be attributed to the weakening of Fe–O bond with As (III) as a result of application of high thermal energy at higher temperature conditions [17,27]. Contrary to As (III), an insignificant impact of temperature on As (V) removal was observed, which may be related to the availability of more Fe adsorption sites (Figure S2A,B) along with strong interactive behavior between HAsO_4_^−2^ and Fe (OH)_2_^+^ under those conditions [2]. Accordingly, kinetic data were also plotted under varying temperatures with results indicating less of a sorption affinity than As (III, V) species at higher temperature conditions (Figure 2B,C). Such an observation is in agreement with our previous study [17], which showed a similar removal trend of Sb (III, V) species at higher temperature. It is noteworthy that As (V) species showed a higher sorption affinity than As (III) species (Figure 2B,C). The sorption kinetics data presented another interesting observation, which was the attainment of equilibrium As (V) concentration in a very short time interval (Figure 2C). Both observations indicate the strong involvement of electrostatic forces of attraction between positively charged Fe species and negatively charged As (V) species at the start of the reaction followed by the adsorption mechanism [2,11].

The sorption kinetics data was well fitted with PFO and PSO models for the removal of As (III) and As (V) species as indicated in Table 2. The R^2^ values for As (III) species at temperatures of 15 °C, 25 °C, and 35 °C by PFO and PSO were 0.994, 0.996, and 0.999 and 0.999, 0.999, and 0.999, respectively. Moreover, R^2^ value of PFO and PSO models for As (V) species were observed to be 0.999 under all temperature conditions, thus indicating a slightly better fitting by pseudo-second order model. The study of temperature and contact time was helpful in understanding the As removal process under such conditions. In general, it was noted that the optimum temperature range for the As (III, V) removal process lies between 15–25 °C. Therefore, further study was conducted at an optimum temperature of 25 °C.

### 3.3. Relationship between FC Dose and As Uptake Index

Figures S3–S5 of SI indicate the FHO formation in solutions containing various As (III, V) oxyanions at concentrations of 100, 500, and 1000 µg/L under applied FC doses after C/F/S process. It has been previously reported [2] that As species significantly interfere with FHO formation at acidic and alkaline pH environments. Therefore, the experiments were conducted in neutral pH conditions with results indicating that FHO formation is a function of applied FC dose and that As species have negligible influence on the FHO formation in solution. Similarly, our previous study [17] shows an increase in FHO formation with increasing FC dose in the presence of antimony oxyanions at neutral pH environments.

**Figure 3:**
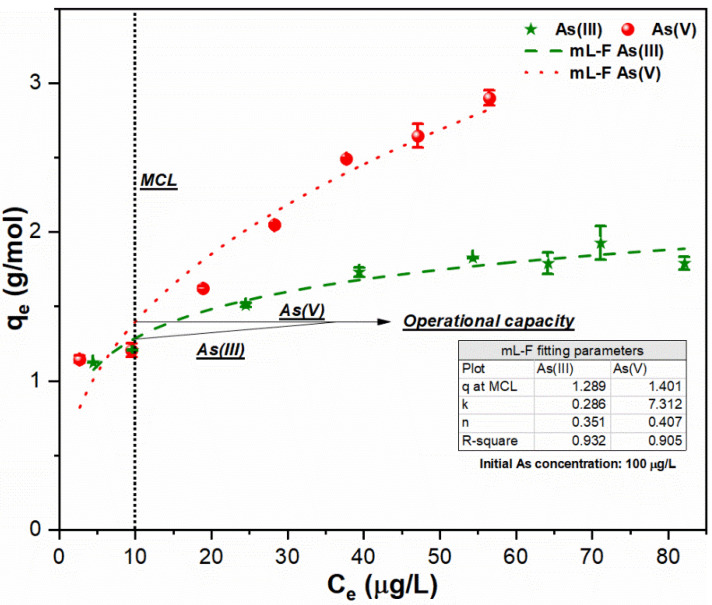
Sorption of As (III, V) species using mL-F model onto FHO surface at conditions: optimum pH of 7; temperature of 25 °C; FC dosage of As (III): 0.01–0.085 mM; As (V): 0.015–0.085 mM; and initial As loading of 100 µg/L.

**Figure 4:**
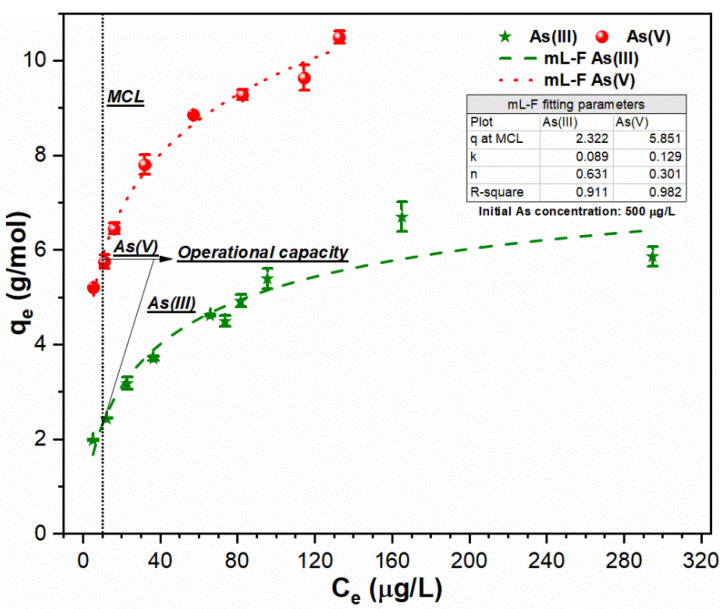
Sorption of As (III, V) species using mL-F model onto FHO surface at conditions: optimum pH of 7; temperature of 25 °C; FC dosage of (As (III): 0.035–0.25 mM; As (V): 0.035–0.095 mM); and initial As loading of 500 µg/L.

**Figure 5:**
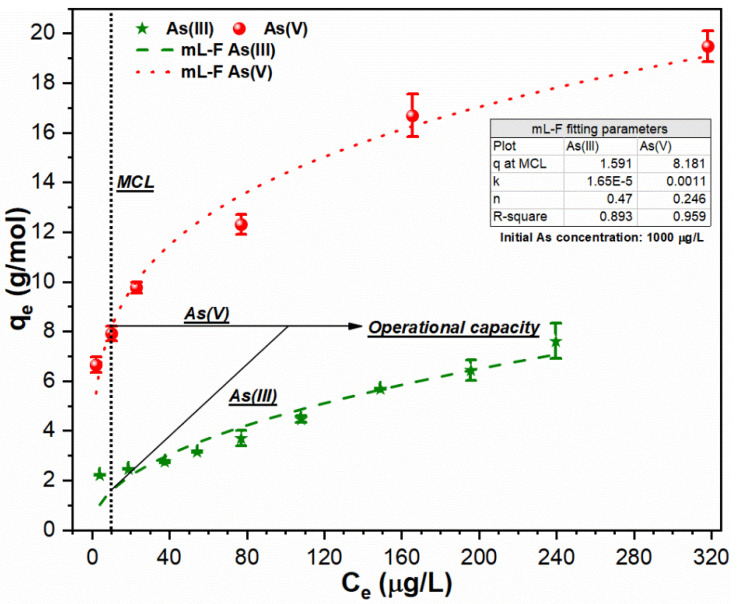
Sorption of As (III, V) species using mL-F model onto FHO surface at conditions: optimum pH of 7; temperature of 25 °C; FC dosage of (As (III): 0.1–0.45 mM; As (V): 0.035–0.15 mM) and initial As loading of 1000 µg/L.

In order to analyze the relationship between sorption affinity and residual As (III, V) concentration, C/F/S experiments were conducted to identify the desired coagulant dose to meet MCL of As species. The sorption data for both As (III, V) species were plotted and corresponding isotherms were fitted using mL-F model (Figure 3, Figure 4 and Figure 5). As shown in Figure 3, the adsorption behavior of As oxyanions with low concentrations (100 µg/L) were better described by mL-F isotherms with higher regression coefficients R^2^ (As (III): 0.932; As (V): 0.905). The low *k_L.F_* value (0.286) was obtained for As (III) species, thus confirming multilayer formation onto heterogeneous FHO sites while the greater *k_L.F_* value (7.312) indicated homogeneous sorption of As (V) species onto FHO surface [28,29,30]. Moreover, the FHO showed similar sorption affinity at MCL (q_MCL_ (As [III]: 1.289 g/mol; As [V]: 1.401 g/mol)) for suspensions containing low As concentration (100 µg/L) in response to applied FC dose. It is worth mentioning that the As (III) uptake may be the same as (V) if the removal process only involved the oxidation of trivalent As and the subsequent sorption of oxidized As species [14]. Based on sorption affinity, residual As concentration and uptake index, the required quantity of FC dose for suspensions with 100 µg/L As (III) Equation (7) and As (V) Equation (8) can be determined as below:

As (III): FC dose (mM) = [100 − 10 µg/L] × [1/10^3^]/[1.289 g As (III)/mol Fe(III)] = 0.069 mM (7)

As (V): FC dose (mM) = [100 − 10 µg/L] × [1/10^3^]/[1.401 g As (V)/mol Fe(III)] = 0.064 mM (8)

For suspensions containing 500 µg/L As (III, V) concentration, the sorption data was also better fitted with mL-F isotherm as indicated by high regression coefficients R^2^ (As (III): 0.911; As (V): 0.982) (Figure 4). The low *k_L.F_* values (As (III): 0.069; As (V): 0.129) indicated that sorption of both toxicants followed Freundlich isotherm [15]. The higher uptake index for both toxicants (q_MCL_ (As [III]: 2.322 g/mol; As [V]: 5.851 g/mol)) were obtained at MCL. The enhanced sorption affinity of As (V) species might be attributable to the effective removal of negatively charged As (V) species (H_2_AsO_4_^−^ and HAsO_4_²^−^) by positively charged FHO (Fe (OH)_2_+) via charge neutralization mechanism [2,8]. The required FC dose for solutions containing 500 µg/L As (III) Equation (9) and As (V) Equation (10) can be obtained using the following equations:

As (III): FC dose (mM) = [500 − 10 µg/L] × [1/10^3^]/[2.322 g As(III)/mol Fe (III)] = 0.211 mM (9)

As (V): FC dose (mM) = [500 − 10 µg/L] × [1/10^3^]/[5.851 g As(V)/mol Fe (III)] = 0.084 mM (10)

The sorption of higher As (III, V) concentration (1000 µg/L) onto FHO also showed good fitting parameters with mL-F isotherm with regression coefficients R^2^ (As (III): 0.893; As (V): 0.959) (Figure 5). The very low *k_L.F_* values (As (III): 0.0000165; As (V): 0.0011) indicate the multilayer sorption properties of both As species on active surface sites of FHO [15]. The much higher uptake index q_MCL_ was obtained for As (V) (8.181 g/mol) as compared to As (III) (1.591 g/mol) at MCL. It is also notable that the uptake index of FHO was reduced in suspensions containing 1000 µg/L As (III) concentration than that observed for 500 µg/L As (III) solutions (Figure 4 and Figure 5). Such observation might be attributed to the fact that As (III) is a weaker Lewis base with a first dissociation constant pka of 9.22, hence a weaker interaction of amphoteric precipitated FHO with As (III) resulted in a smaller uptake index [24]. The required FC dose for solutions containing 1000 µg/L As (III) Equation (11) and As (V) Equation (12) is given as below:

As (III): FC dose (mM) = [1000 − 10 µg/L] × [1/10^3^]/[1.591 g As (III)/mol Fe (III)] = 0.622 mM (11)

As (V): FC dose (mM) = [1000 − 10 µg/L] × [1/10^3^]/[8.181 g As (V)/mol Fe (III)] = 0.121 mM (12)

Based on above results, it was identified that a higher As (III) concentration (1000 µg/L) consumes a greater quantity of FC dose (0.622 mM), which ultimately will increase sludge handling issues as well as increase the operating cost. Therefore, our current study suggests two-stage C/F/S as an effective process in the removal of higher concentration of As (III) species from water. This may involve: (1) a coagulation-flocculation (C/F) of 1000 µg/L As (III) with FC dose of 0.2 mM, thus lowering As (III) concentration to 107.9 µg/L; (2) a coagulation-flocculation of supernatant-containing As (III) from first step with FC dose using Equation (7), which will ultimately decrease the As (III) concentration below MCL and; (3) a final sedimentation process followed by filtration using 0.45 micron filter. Such a useful approach may not only be helpful in meeting the regulatory limit of As (III) species, but also significantly decrease the quantity of required coagulant dose. For instance, the current study remarkably lowers the required FC dosage from 0.622 to 0.269 m mol/L (around 231%) for As (III) removal from aqueous environment. Although, previous studies [24,29] suggest oxidation followed by adsorption as an effective removal strategy for As (III) species, our current study proposes the feasible two-step C/F/S process for treating water containing higher concentration of As (III) species.

### 3.4. Application of Experimental Strategy on Simulated Water Sample

In order to obtain accurate results at laboratory scale, it is important to adjust experimental conditions similar to the natural water environment. A natural waters matrix, such as the National Sanitation Foundation (NSF) standard was therefore used as reference for evaluating the As removal potential using our calculated FC dose. The desired concentrations (100, 500 and 1000 µg/L) of As (III, V) species were spiked in an NSF waters matrix to evaluate the effectiveness of our experimental approach. The results indicate that residual As (III) concentrations of 55, 273, and 427 µg/L were achieved for NSF water samples spiked with pollutant loading of 100, 500, and 1000 µg/L, respectively. While the residual As (V) concentrations were found to be 41, 189, and 284 µg/L for initial contaminant loading of 100, 500, and 1000 µg/L, respectively. From these findings, it is evident that a relatively higher quantity of FC dose is required in real water samples owing to competitive and inhibitory effects of various cations (Ca^2+^, Mg^2+^, Na^+^) and anions (HCO_3_^−^, Cl^−^, SO_4_^2^^−^, SiO_2_^−^, NO_3_^−^, F^−^, PO_4_^3^^−^). It was observed that the presence of divalent cations significantly enhance the As removal as compared with monovalent cations during the C/F/S process [11]. Another study indicates the inhibitory effect of anions on As removal performance, showing competitive inhibition by SO_4_^2^^−^ for sorption sites of FHO. The presence of other anions i.e., PO_4_^3^^−^ and SiO_2_^−^ had a detrimental effect on coagulation performance owing to their role in sequestering the FHO formation along with competitive adsorption onto FHO surface [31]. The effect of each individual interfering species on As (III, V) removal is mentioned in detail elsewhere [11,31,32]. In order to achieve MCL for 100, 500, and 1000 µg/L As (III, V) in an NSF waters matrix, experimental results indicate a required FC dose of 0.19, 0.56, and 2.18 mM for As (III) suspensions while 0.125, 0.25, and 0.60 mM for As (V) solutions, respectively. Conclusively, the experimental results of the study verify that the suggested process can minimize initial As concentration below the drinking water regulation limit, as well as the q_MCL_ value. Therefore, future studies on C/F/S performance should also include a natural water matrix to further understand the removal mechanism of other toxicants during water treatment operations.

### 3.5. Environmental Implications

In order to examine the efficacy of water treatment system, knowledge about the operating conditions such as pH, temperature, initial contaminant loading and other suspension parameters contains significant importance. The arsenic pollution in drinking water facilities has already pose a great threat to human health and ecosystem. The current study is an attempt to optimize the C/F/S process conditions with main aim of lowering As (III, V) concentration from water below the MCL level. Our results also highlighted that the reduced form of As species will be more dominant in aqueous solution owing to their less affinity towards FHO species, thus posing 10 times more threat to humans as compared to its oxidized form. Adding more, pH and temperature has a detrimental impact on As (III) removal efficiency with optimum pH range 7–10 and low temperature conditions. Thus environmental water matrix with pH above 7 at low temperature has the advantage that the trivalent contaminants are favorably eliminated from an aqueous media. In addition, current study provides the importance of developing uptake indices for As oxyanions by FC in water. Such strategy will be helpful in achieving MCL level of both toxicants, thereby reducing the ecotoxicological risks especially in environments contaminated with As (III) species. The present study may be helpful in predicting the mobility, transport and fate of As species in the aquatic environment, thus minimizing the associated health risks.

## 4. Conclusions

This work systematically examined the effect of various operating parameters such as pH, contact time, temperature, As (III, V) concentration, ferric chloride (FC) dose, and interfering ions on the removal performance of As oxyanions during the C/F/S process. If the source water pH is set as 7, effective As (III, V) removal can be achieved with greater sorption affinity of pentavalent species than trivalent from water. The high temperature conditions of suspensions containing As oxyanions will remarkably decrease the removal performance of both toxicants from water. The kinetic study showed a better fitting of sorption data with pseudo-second order model, while isotherm studies presented excellent fitting with modified Langmuir–Freundlich model. Under optimum pH and temperature, further study addresses the effectiveness of the C/F/S process in achieving MCL for As oxyanions in water. The uptake index (q_MCL_) was developed to select an FC dose that helps meeting drinking water regulation limits for As (III, V) species. The results show that the removal performance of the C/F/S process depends on the initial concentration of pollutant, where high As contamination levels require greater FC dose. Moreover, the presence of interfering species resulted in an increase in the required FC dose for achieving MCL in a real water environment. Following the excellent removal capability of FC coagulant in a variety of water treatment applications, future research efforts should be focused on developing uptake indices under heterogeneous aquatic conditions to minimize associated human health risks.

## Figures and Tables

**Table 1:** Ion concentrations in the NSF simulated water matrix.

Cations	mg/L	Anions	mg/L
Na^+^	88.8	HCO_3_^−^	138
Ca^2+^	40.0	SO_4_^2^^−^	50
Mg^2+^	12.7	Cl^−^	71
		NO_3_^−^	2
		F^−^	1
		PO_4_^3^^−^	0.04
		SiO_2_	22.4

**Table 2:** Sorption kinetic parameters of trivalent and pentavalent As ions on precipitated FHO under various temperatures (15–35 °C).

		PFO			PSO		
Ions	Temperature	*k*_1_ (1/min)	*q_e_* (g/mol)	R^2^	*k*_2_ (mol)/g min)	*q_e_* (g/mol)	R^2^
As (III)	15 °C	0.414	7.758	0.994	0.089	8.332	0.999
	25 °C	0.504	7.575	0.996	0.132	7.996	0.999
	35 °C	0.654	5.603	0.999	0.322	5.789	0.999
As (V)	15 °C	0.954	9.585	0.999	0.551	9.701	0.999
	25 °C	0.808	9.505	0.999	0.342	9.685	0.999
	35 °C	1.294	9.074	0.999	1.506	9.123	0.999

## Data Availability

All data used to support the findings of this study are included within the article.

## Supplementary Materials

The following are available online at https://www.mdpi.com/article/10.3390/ijerph18189812/s1, Figure S1: (A) FHO formation in the presence of 1000 µg/L As (III,V) species, pH range of 4–10, temperature of 25 °C, and FC dosage of 0.1 m mol/L; (B, C) Speciation diagrams of Fe (III), As (III) and As (V) species using chemical modelling software Visual MINTEQ, Figure S2: FHO formation in the presence of 1000 µg/L (A) As (III) and (B) As (V) species, neutral pH, 0.1 m mol/L FC dosage and various temperature (15–35 °C) and contact times (0–28 min), Figure S3: FHO formation in the presence of 100 µg/L (A) As (III); and (B) As (V) species at optimum pH and temperature under various FC dosages in water, Figure S4: FHO formation in the presence of 500 µg/L (A) As (III); and (B) As (V) species at optimum pH and temperature under various FC dosages in water, Figure S5: FHO formation in the presence of 1000 µg/L (A) As (III); and (B) As (V) species at optimum pH and temperature under various FC dosages in water.
