# Supplementary material for: Removal of Arsenic Oxyanions from Water by Ferric Chloride—Optimization of Process Conditions and Implications for Improving Coagulation Performance
Source: Int J Environ Res Public Health. 2021 Sep 17;18(18):9812. doi: 10.3390/ijerph18189812 (PMC8465526; doi:10.3390/ijerph18189812)
Supplement: Supplementary file 1 [file ijerph-18-09812-s001.zip › ijerph-1367843-supplementary.pdf]

*Supplementary Information*

# Removal of Arsenic Oxyanions from Water by Ferric Chloride—Optimization of Process Conditions and Implications for Improving Coagulation Performance

**Muhammad Ali Inam [1], Rizwan Khan [2], Kang Hoon-Lee [3,*] and Young Min-Wie [4]**

[1] Institute of Environmental Sciences and Engineering (IESE), School of Civil and Environmental Engineering (SCEE), National University of Sciences and Technology (NUST) H-12 Campus, Islamabad 44000, Pakistan; ainam@iese.nust.edu.pk

[2] Department of Chemical Engineering, Quaid-e-Awam University of Engineering, Science and Technology (QUEST), Nawabshah, Sindh 67480, Pakistan; rizwansoomro@quest.edu.pk

[3] Department of Civil and Environmental Engineering, Hanyang University, 222 Seongdong-gu, Seoul 04763, Korea

[4] Department of Materials Engineering, Kyonggi University, Suwon, 162-27, Korea supreme98@kyonggi.ac.kr

* Correspondence: diasyong@hanyang.ac.kr

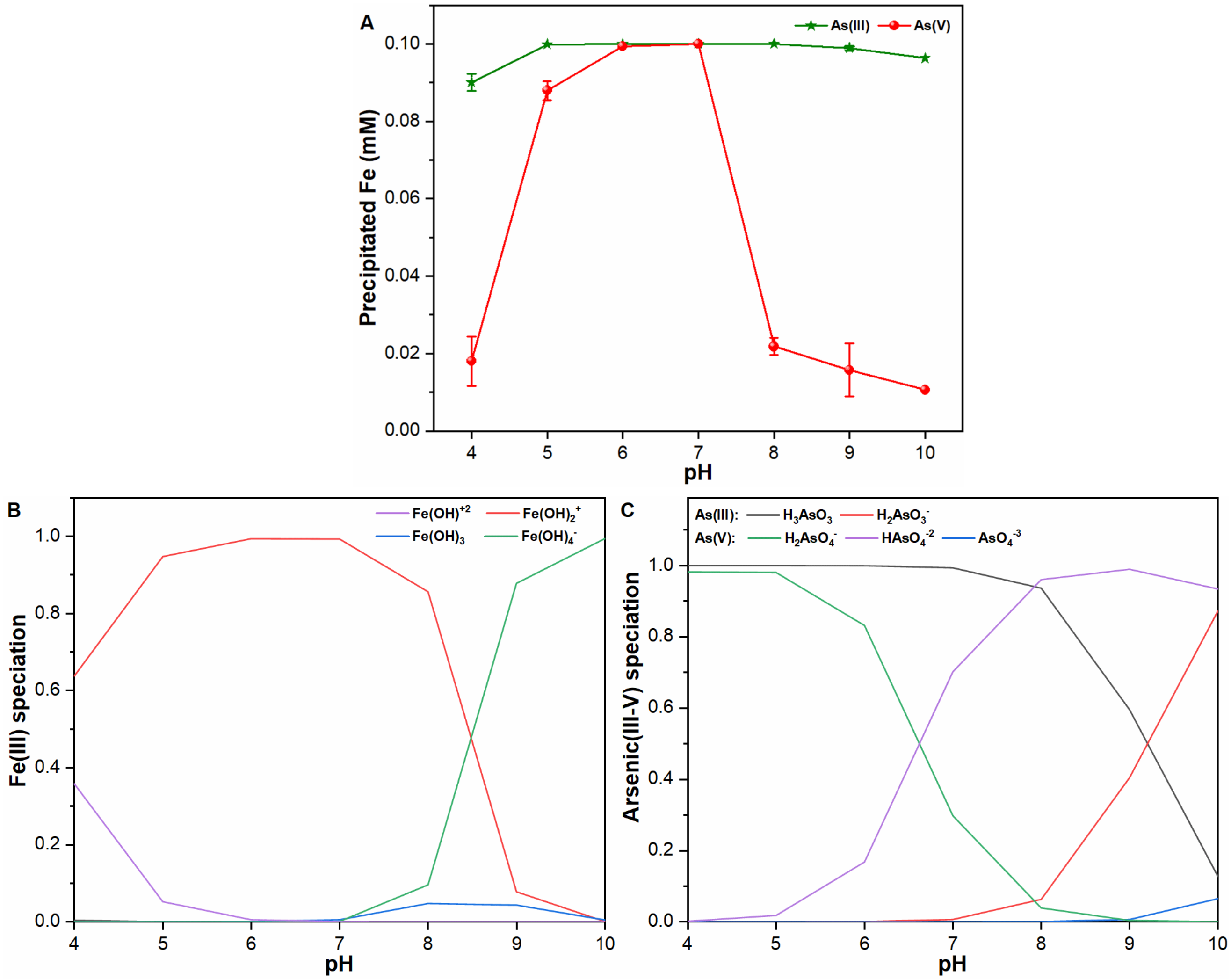


**Figure S1.** (**A**) FHO formation in the presence of 1000 μg/L As (III–V) species, pH range of 4–10, temperature (25 °C) and FC dosage of 0.1 m.mol/L; (**B**,**C**) Speciation diagrams of Fe (III), As (III) and As (V) species using chemical modelling software Visual MINTEQ.

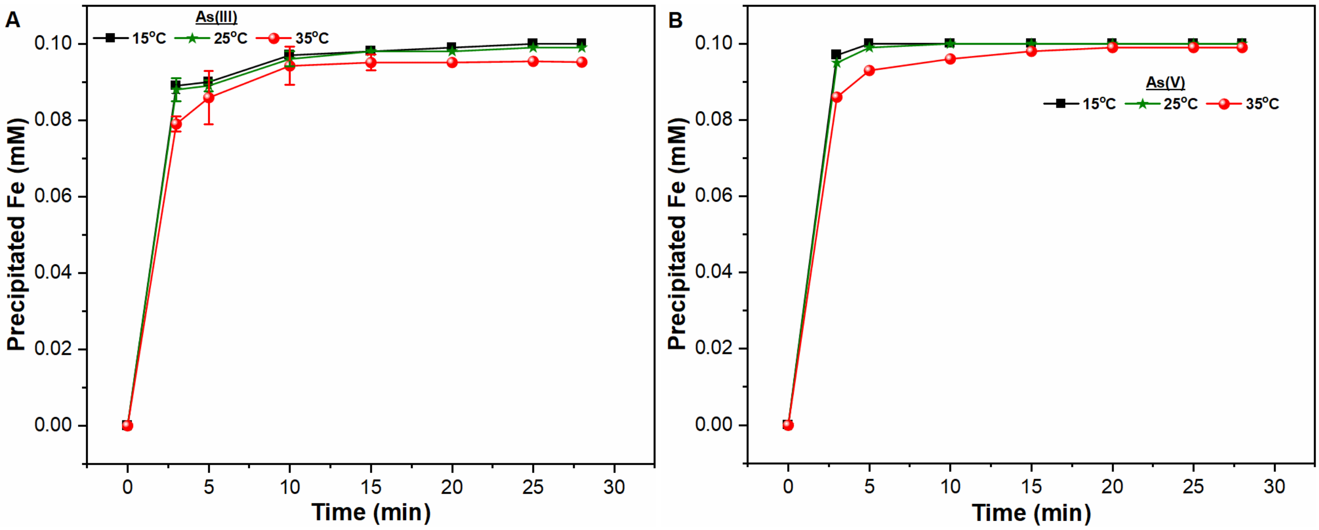


**Figure S2.** FHO formation in the presence of 1000 μg/L (**A**) As (III) and (**B**) As (V) species, neutral pH, 0.1 m.mol/L FC dosage and various temperature (15–35°C) and contact time (0–28 min).

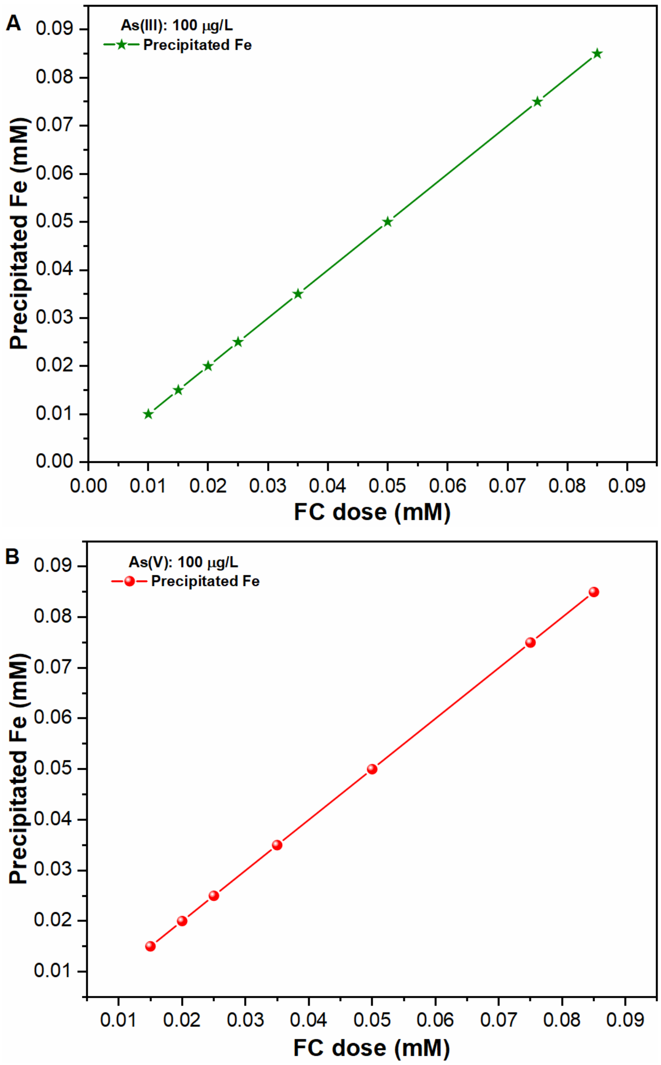


**Figure S3.** FHO formation in the presence of 100 μg/L (**A**) As (III); and (**B**) As (V) species at optimum pH and temperature under various FC dosages in water.

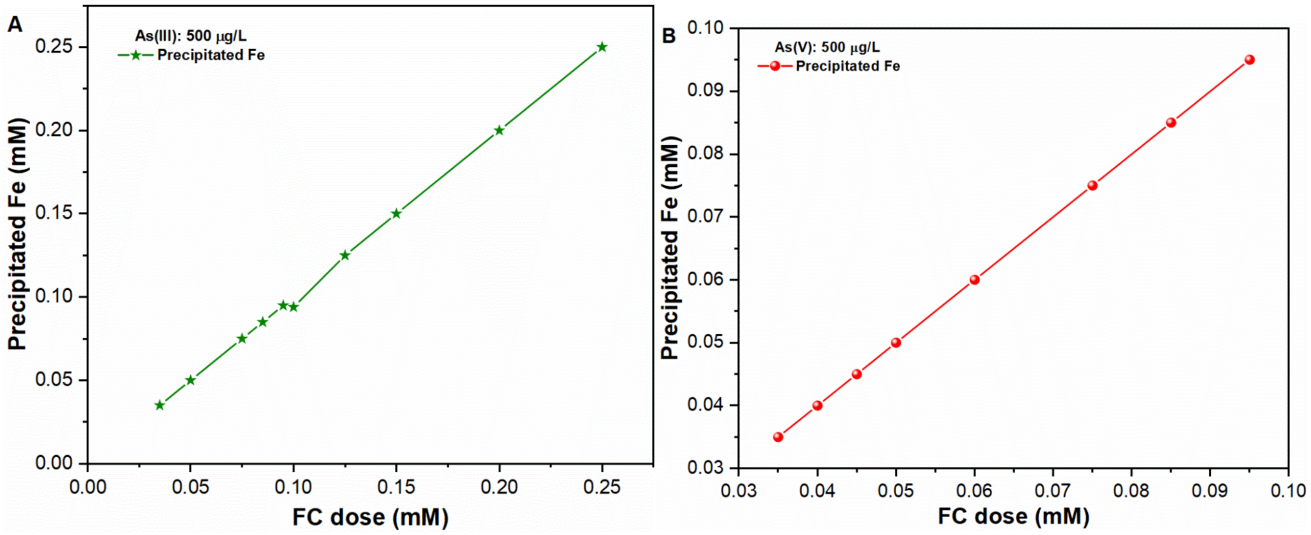


**Figure S4.** FHO formation in the presence of 500 μg/L (**A**) As (III); and (**B**) As (V) species at optimum pH and temperature under various FC dosages in water.

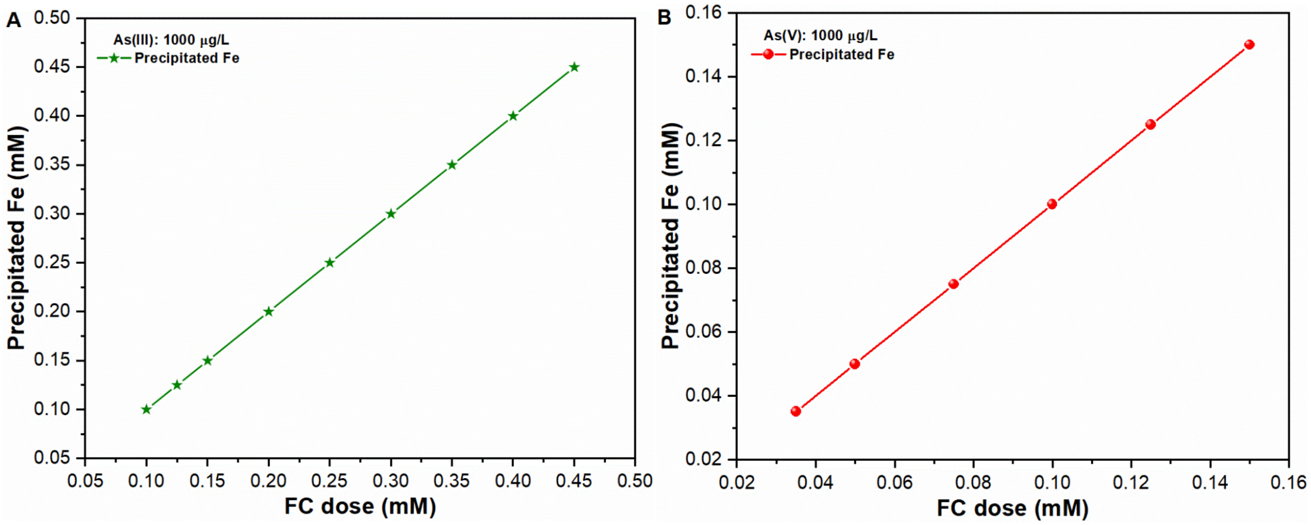


**Figure S5.** FHO formation in the presence of 1000 μg/L (**A**) As (III); and (**B**) As (V) species at optimum pH and temperature under various FC dosages in water.
